# Yogurt consumption is associated with higher nutrient intake, diet quality and favourable metabolic profile in children: a cross-sectional analysis using data from years 1–4 of the National diet and Nutrition Survey, UK

**DOI:** 10.1007/s00394-017-1605-x

**Published:** 2018-01-12

**Authors:** D. A. Hobbs, D. I. Givens, J. A. Lovegrove

**Affiliations:** 1 Hugh Sinclair Unit of Human Nutrition, Department of Food and Nutritional Sciences, University of Reading, Whiteknights, Reading, RG6 6AP UK; 2 Institute for Food, Nutrition and Health, University of Reading, Reading, UK; 3 Institute for Cardiovascular and Metabolic Health, University of Reading, Reading, UK

**Keywords:** Cardiometabolic health, Diet quality, Dietary patterns, Nutrient intakes, Yogurt

## Abstract

**Purpose:**

Yogurt consumption has been associated with higher nutrient intakes, better diet quality and improved metabolic profiles in adults. Few studies have investigated these associations in children. This study investigated the association of yogurt consumption with nutrient intakes, diet quality and metabolic profile in British children.

**Methods:**

Data from  1687 children aged 4–10 and 11–18 years of the National Diet and Nutrition Survey (NDNS) years 1–4 were analysed. Yogurt consumption was determined using a 4-day diet diary. Diet quality was assessed by the Healthy Eating Index 2010 (HEI-2010). Anthropometric measures, blood pressure, pulse pressure, plasma glucose, HbA1c, C-reactive protein, triacylglycerol, total cholesterol, high-and low-density cholesterol from NDNS were used.

**Results:**

The highest tertile of yogurt consumption (T3) was associated with higher nutrient intakes, particularly for calcium (children 4–10 years: *P* < 0.0001; children 11–18 years *P* = 0.001), iodine (both age groups *P* < 0.0001) and riboflavin (both age groups *P* < 0.0001), and HEI-2010 score (both age groups *P* < 0.0001) in children aged 4–10 years (mean ± SD: 98.4 ± 35.7 g yogurt/day) and 11–18 years (mean ± SD: 105.4 ± 37.5 g yogurt/day) compared with non-consumers (0 g yogurt/d). Yogurt consumption was associated with significantly lower pulse pressure in children aged 4–10 years and lower HbA1c concentration, being shorter and having a larger hip circumference in children aged 11–18 years, compared with non-yogurt consumers.

**Conclusion:**

This study suggests that British children who are yogurt consumers (> 60 g/day) have higher overall diet quality, nutrient intakes and adequacy, lower pulse pressure (children aged 4–10 years) and HbA1c concentrations (children aged 11–18 years), were shorter and had a smaller hip circumference (children aged 11–18 years).

**Electronic supplementary material:**

The online version of this article (10.1007/s00394-017-1605-x) contains supplementary material, which is available to authorized users.

## Introduction

Dairy products are a good source of a number of important nutrients in the British diet such as energy, protein and other macro- and micronutrients. However, dairy fats are high in saturated fat, and it is estimated that dairy products (excluding butter) contribute 31% and 22% of saturated fat in the diets of British children aged 4–10 and 11–18 years, respectively [1]. High intakes of saturated fat have been associated with an increased risk of coronary heart disease (CHD), thus the majority of recommendations to reduce risk of CHD have focused on the reduction of saturated fat in the diet. In contrast, consumption of dairy products is not associated with an increased risk of CVD [2], and evidence suggests dairy product consumption is associated with a reduced risk of metabolic syndrome [3, 4].

There is an increasing body of epidemiological and clinical evidence to suggest that yogurt consumption may be beneficial to cardiometabolic health. For example, data suggest that yogurt consumption is associated with a smaller common carotid artery intima-media thickness [5], reduced risk of colorectal cancer [6] and type 2 diabetes [7–10]. Furthermore, yogurt consumption has been shown to be inversely associated with long-term bodyweight gain [11] as well as improved nutrient intakes [12]. However, not all studies have found a beneficial effect of yogurt consumption on health. For example, the study by Sayon-Orea et al. [13] found that there was no effect of yogurt consumption on risk of developing metabolic syndrome over a 6-year period in adults. However, the authors did report a significant inverse effect of central adiposity with the highest yogurt intake. To date very few studies have investigated the possible effects of yogurt consumption in younger populations. One study that did, was that by Zhu et al. [14] who examined the associations between yogurt consumption and metabolic profile in American children using data from the National Health and Nutrition Examination Survey (NHANES) and found that frequent yogurt consumers had a better diet quality and insulin profile. In addition, using data from years 1–3 of the UK National Diet and Nutrition Survey (NDNS), Williams et al. [12] found that yogurt consumption made an important contribution to nutrient intakes in UK diets, particularly in children. Collectively these data suggest that yogurt is an important dietary component that may increase nutritional adequacy and improve metabolic profile in children, although more evidence is needed for confirmation. Only one study has assessed the nutrient contents of diets containing yogurt, using data from years 1–3 of the NDNS [12]. To date few studies have investigated the effects of yogurt consumption on metabolic profile in children. Therefore, there is a need to examine the association between yogurt consumption and nutrient intakes, diet quality and metabolic profile in children using years 1–4 of the NDNS. The additional strength of this paper is that it uses data from years 1–4 of the NDNS, which includes more recent data (collected from 2009/10–2011/12), more participants as well as more consumers of yogurt compared with previous studies using data from years 1–3 of the NDNS [12]. This paper uses data from British children, but would have implications for children in other EU countries, although the dietary patterns may differ, analysis in other populations would be advisable.

The aim of the current study was to investigate the association between yogurt consumption, nutritional adequacy, diet quality, and metabolic and health biomarkers in children (4–18 years) using data from years 1–4 of the NDNS, UK.

## Methods

### Study population

Participants in this analysis were 1687 children aged 4–18 years from years 1–4 of the NDNS rolling programme (2008/2009–2011/2012), the methodology of which has been described in detail elsewhere [1].

Briefly, the NDNS is a cross-sectional survey of the food consumption, nutritional intakes and nutritional status of people aged 1.5 years and older living in private households in the UK. Randomly selected participants were invited to complete a 4-day food diary, interviewed and asked to provide a fasted blood and urine sample. The 4-day estimated food diary was completed on 4 consecutive days and the start dates for the 4-day food diaries were randomized to get a representative sample of all days of the week. For children aged 11 years or younger, the parent/carer was asked to complete the 4-day food diary with assistance from the child. Children aged 12 years and above were asked to complete the 4-day food diary themselves. In each case participants were asked to describe their food portions using standard household measures. Participants also completed a computer based personal interview, collecting information on dietary habits and lifestyle, such as household information, information on circumstances/habits that could affect their food intake, employment status, educational background and household income. Participants also had their height and weight measured, from which BMI was calculated. In a follow-up household visit by a nurse, waist:hip ratio and blood pressure was measured, and a fasted blood sample was taken for those aged 4 years and above. The blood samples were analyzed for a number of analytes, including total cholesterol, high-density lipoprotein (HDL) cholesterol, low-density lipoprotein (LDL) cholesterol, triacylglycerols (TAGs), C-reactive protein (CRP), glycated haemoglobin (HbA1c) and glucose. The assays used for measurement of each analyte have been published previously [15]. Nutrient intakes from the 4-day diet diaries were calculated using the NDNS databank, which is based on McCance and Widdowson’s Composition of Foods series and the FSA’s Food Portion Size guides. The databank is updated annually and also includes data from manufacturers, as it contains nutritional information given on food labels. The nutrient intakes reported in this analysis come from foods consumed and do not include nutrients from vitamin or mineral supplements.

The NDNS was conducted according to the guidelines laid down in the Declaration of Helsinki, and ethical approval for all procedures was granted by Local Research Ethics Committees covering all areas in the survey. All participants (or where relevant legal guardians) gave informed consent.

### Estimation of yogurt consumption

Yogurt intakes were calculated based on the average weight of yogurt consumed per day from the 4-day diet diary. In this analysis the yogurt food group was defined as all yogurts (fat free, low-fat and high-fat, including Greek yogurt) and fromage frais (a type of smooth soft fresh cheese with the consistency of thick yogurt). Dairy desserts were excluded from the food group. A non-consumer of yogurt was defined as an individual who did not consume any yogurt or fromage frais during any of the four days of dietary recording.

### Assessment of diet quality

Diet quality was measured using the Healthy Eating Index 2010 (HEI-2010) with some modifications, which were based on the 2007 dietary guidelines for UK institutions [16]. (Supplemental Methods 1 and Supplemental Table 1). It was decided to use HEI because it is the most suited indicator of diet quality of children’s diets and has been used previously in this population [14]. A description of the methodology has been described in detail previously [17] and steps for calculating HEI-2010 are also available from the National Cancer Institute [18] and the National Institute on Ageing [19]. Briefly, the HEI-2010 scores 12 food groups/components for a total of 100 points, with a maximum score being achieved if diets meet recommendations for a particular food group/component, and a lower score assigned if diets do not meet recommendations. Six of the components (total fruit, whole fruit, total vegetables, greens and beans, seafood and plant proteins, and total protein foods) were worth 0–5 points, 5 components (whole-grains, dairy [included milks, cheese, yogurt, fromage frais, dairy desserts], fatty acids ratio [(PUFA + MUFA/SF)], refined grains and sodium) were worth 0–10 points, and one component (empty calories, which included energy from solid fats, added sugars) was worth 0–20 points and is reverse scored, such as a lower intake was given a better score. Refined grains and sodium were also reverse scored. The same dietary guidelines were applied to both age groups, apart from for sodium, which was scored according to age (4–6, 7–10 and > 11 years), because UK sodium guidelines are different for these age groups. All components except the fatty acids ratio were scored on a density basis (per 1000 kcal) or as a percentage of total energy.

## Assessment of under-reporting

Misreporting is a well-documented issue in self-reported dietary methods [20, 21]. The methods used to determine energy misreporting in children and adolescents have been described in detail previously [22]. Briefly, energy misreporting was assessed using Goldberg’s cut-off2 criterion [23, 24], which uses 95% confidence limits to statistically compare the ratio of reported energy intake (EI) to basal metabolic rate (BMR) with physical activity level (PAL). PAL was adapted to children and corresponded to light physical activity (1.45–1.60 depending on age and gender) [22]. BMR was estimated according to Schofield’s equations [25] taking into account age, gender and height of the subjects [26]. The within-subject variation in reported energy intake (CV_wEI_) was 24% for children aged < 6 years and 23% for the others [27]. The within-subject variation in repeated BMR measurements (CV_wBMR_) was 8.2% as recommended by Black and Cole [28]. The between-subject variation in PAL (CV_tP_) was 15% [23]. Subjects were identified as under-reporters if their reported EI was less than the calculated lower cut-off.

### Data and statistical analysis

For all analysis, participants were organized into non-yogurt consumers (0 g yogurt/day) and yogurt consumers (> 0 g yogurt/day). Yogurt consumers were further divided into tertiles of yogurt consumption with tertile 1 (T1) being the lowest yogurt consumers and tertile 3 (T3) being the highest yogurt consumers. In addition, data were split into two age groups (4–10 and 11–18 years) to be in line with the NDNS usual classification [1], but also because dietary habits and nutrient recommendations are generally different between the two age groups as shown in the study by Williams et al. [12]. Furthermore, participants with CRP above 10 mg/L were removed from the analysis as this is indicative of possible infection/illness.

Analysis of covariance (ANCOVA), adjusted for age, sex and total energy intake (kJ) was used to determine differences in nutrient intakes, food groups, HEI-2010 index scores and biomarkers of metabolic health between non-consumers and tertiles of yogurt consumption. The statistical analyses of biomarkers of metabolic health were further adjusted for HEI-2010 score (model 3). There was no association between BMI and HEI in children aged 4–10 years (*P* = 0.728) and children aged 11–18 years (*P* = 0.845). In addition, Bonferroni pairwise tests were used to detect differences between non-consumers and tertiles. Differences in sociodemographic characteristics between non-consumers and tertiles of yogurt intake were determined using Chi-square test for independence. Sensitivity analysis was performed after excluding subjects identified as under-reporters in children aged 4–10 (*n* = 114, 15%) and 11–18 (*n* = 405, 47%) years to ensure the results were not biased. All statistical analysis was performed in SPSS for Windows 20 (SPSS Inc., Chicago, IL, USA). *P* values of < 0.05 were considered statistically significant.

## Results

In this population (*n* = 1687) 38% of children aged 4–10 years and 69% of children aged 11–18 years did not consume any yogurt during the 4 days of recording. The mean (± standard deviation) yogurt consumption ranged from 19 ± 8 g yogurt/day in tertile 1 to 98 ± 36 g yogurt/day in tertile 3 for 4–10 year olds and 20 ± 8 g yogurt/d in tertile 1 to 105 ± 36 g yogurt/day in tertile 3 for 11–18 year olds (Table 1). Yogurt was most commonly consumed at lunch (32%), followed by dinner (18%), breakfast (14%), evening snack (13%), afternoon snack (12%) and morning snack (11%) for children aged 4–10 years and for children aged 11–18 years yogurt was most commonly consumed at lunch (26%), dinner (18%), evening snack (18%), morning snack (14%), breakfast (13%) and afternoon snack (11%). There were no significant differences in study characteristics between non-consumers and across increasing tertiles of yogurt intake in children aged 4–10 y, but for children aged 11–18 years there was a significant difference in age (*P* < 0.0001), with the non-consumers being significantly older than tertile 1 (*P* < 0.0001), tertile 2 (*P* = 0.001) and tertile 3 (*P* = 0.003).

**Table 1:** Study characteristics of British children aged 4–10 and 11–18 years (*n* = 1687) from years 1–4 of the NDNS for non-yogurt consumers and according to tertiles of yogurt and fromage frais consumption

Sociodemographic characteristics	Children 4–10 years Yogurt tertiles				*P* ^a^	Children 11–18 years Yogurt tertiles				*P*
	NC (*n* = 307)	T1 (*n* = 166)	T2 (*n* = 155)	T3 (*n* = 175)		NC (*n* = 610)	T1 (*n* = 97)	T2 (*n* = 89)	T3 (*n* = 88)	
Yogurt consumption, g/d	0 ± 0	19.4 ± 7.8	43.2 ± 8.3	98.4 ± 35.7		0 ± 0	19.8 ± 7.8	40.9 ± 8.3	105.4 ± 37.5	
Age, years	7.1 ± 2.1	6.9 ± 2.0	6.7 ± 2.0	7.0 ± 2.0	0.17	14.8 ± 2.1	13.7 ± 2.2	13.9 ± 2.4	14.0 ± 2.3	0.0001
Sex, %					0.58					0.29
Male	51.5	51.5	47.7	55.1		50.0	43.3	55.1	55.7	
Female	48.5	48.3	52.3	44.9		50.0	56.7	44.9	44.3	
Ethnicity, %					0.12					0.42
Asian or Asian British	5.5	10.8	3.2	4.0		4.4	7.2	3.4	2.3	
White	83.1	76.5	90.3	87.5		88.0	82.5	91.0	90.9	
Black or Black British	5.9	1.8	3.2	1.7		3.3	3.1	3.4	1.1	
Mixed	3.6	6.6	2.0	4.0		1.8	5.1	0.0	3.4	
Any other	2.0	4.3	1.3	2.8		2.5	2.1	2.2	2.3	
NS-SEC8^b^, %					0.07					0.12
Higher managerial/professional	14.0	12.7	18.7	17.0		12.6	18.6	12.4	22.7	
Lower managerial/professional	27.4	21.7	31.0	32.4		27.5	27.8	25.8	20.5	
Intermediate occupations	6.8	7.8	11.0	5.7		7.4	8.2	15.7	6.8	
Small employers/own accounts	10.4	15.7	8.4	8.5		10.5	12.4	7.9	12.5	
Lower supervisory/technical	8.1	13.9	8.4	11.9		9.8	9.3	10.1	13.6	
Semi-routine occupations	15.3	13.9	11.0	13.1		15.4	8.2	13.5	12.5	
Routine occupations	12.1	8.4	8.4	7.4		10.5	10.3	11.2	8.0	
Never worked	4.6	3.6	0.6	1.7		3.8	4.1	2.2	0.0	
Other	1.3	2.4	2.6	2.3		2.5	1.0	1.1	3.4	
Income, %					0.50					0.47
£100,000 or more	3.9	3.6	3.9	4.5		2.3	5.2	2.2	5.7	
£50,000–£99,999	13.7	11.4	16.8	25.1		16.1	19.6	18.0	20.5	
£40,000–£49,999	11.1	12.0	9.7	10.8		8.5	15.5	10.1	10.2	
£30,000-£39,999	16	15.1	21.9	14.2		15.4	12.4	20.2	17.0	
£20,000–29,999	11.4	10.2	14.2	11.9		10.5	6.2	9.0	14.8	
£5,000–£19,999	28	28.4	21.3	21.0		29.3	26.8	28.1	19.3	
Under £5,000	3.9	3.0	3.2	2.8		3.3	3.1	1.1	0.0	
Don’t know/refused	12	16.3	9.0	9.7		14.6	11.3	11.2	12.5	

All values are means ± SDs or percentages

*NC* non-consumer, *NDNS* National Diet and Nutrition Survey, *NS-SEC8* National Statistics Socio-economic classification, *T* tertile

^a^*P* value for difference between categorical variables NC and yogurt tertiles based on Chi-square test for independence

^b^Data for NS-SEC8 and income are based on parent/carer data [29]

## Nutrient intakes and adequacy

Yogurt consumers had significantly different intakes of some nutrients compared with non-consumers (Table 2 and Supplemental Table 2). For children aged 4–10 years there were significant differences across non-consumers and tertiles of yogurt intake when controlling for total energy intake (kJ), age and sex for protein, total fat, monounsaturated fatty acids (MUFAs), n-6 fatty acids, starch, total sugars, intrinsic and milk sugars, lactose, thiamin, riboflavin, vitamin B_12_, sodium, potassium, calcium, magnesium, phosphorus, zinc, iodine, selenium and energy (controlled for age and sex only), with children in the highest tertile (T3) of yogurt intake having significantly (*P* < 0.05) higher intakes of energy, protein, total sugars, intrinsic and milk sugars, lactose, thiamin, riboflavin, vitamin B_12_, potassium, calcium, magnesium, phosphorus, zinc, iodine and selenium compared with non-consumers. In addition, children in the highest tertile (T3) of yogurt intake had significantly (*P* < 0.05) lower intakes of fat, MUFAs, n-6 fatty acids, sodium and starch compared with non-consumers. For children aged 11–18 years there were significant differences across non-consumers and tertiles of yogurt intake for energy, total fat, MUFAs, n-6 fatty acids, carbohydrate, total sugars, intrinsic and milk sugars, fructose, lactose, vitamin A, beta-carotene, thiamin, riboflavin, folate, vitamin C, vitamin D, potassium, calcium, magnesium, phosphorus, iron, zinc, manganese, iodine and selenium, with children in the highest tertile (T3) of yogurt intake having significantly (*P* < 0.05) higher intakes of these nutrients compared with non-consumers, apart from total fat, MUFAs and n-6 fatty acid intakes, which were significantly (*P* < 0.05) lower in the highest tertile (T3) of yogurt intake compared with non-consumers.

**Table 2:** Nutritional adequacy for diets of non-yogurt consumers and across increasing tertile of yogurt and fromage frais consumption using data from years 1–4 of the NDNS for children aged 4–10 and 11–18 years old

Nutrients per day	Children 4–10 years Yogurt tertiles (g/day)				*P* ^a^	Children 11–18 years Yogurt tertiles (g/day)				*P*
	NC (0)	T1 (1–30)	T2 (31–60)	T3 (61–295)		NC (0)	T1 (2–30)	T2 (31–60)	T3 (61–236)	
Participants, *n*	307	166	155	175		610	97	89	88	
Energy, kJ	6150 ± 73	6400 ± 100	6510 ± 104*	6760 ± 97*	0.000	7370 ± 81	8170 ± 2230*	7450 ± 200	8030 ± 205*	0.001
Protein, g	53.7 ± 0.5	53.9 ± 0.6	55.2 ± 0.7	57.1 ± 0.6*	0.0001	65.3 ± 0.5	66.0 ± 1.6	65.4 ± 1.3	69.3 ± 1.4*	0.07
Total fat, g	57.9 ± 0.4	57.4 ± 0.6	56.5 ± 0.6	55.3 ± 0.6*	0.005	68.5 ± 0.4	67.0 ± 1.1	64.3 ± 1.0*	64.3 ± 1.0*	0.0001
MUFA, g	20.9 ± 0.2	20.5 ± 0.3	20.2 ± 0.3	19.5 ± 0.3*	0.0001	26.0 ± 0.2	24.9 ± 0.6	23.6 ± 0.5*	23.4 ± 0.5*	0.0001
n-6 fatty acids, g	7.8 ± 0.1	7.5 ± 0.2	7.4 ± 0.2	6.9 ± 0.2*	0.0001	9.4 ± 0.1	9.1 ± 0.3	9.0 ± 0.3	8.6 ± 0.3*	0.030
Carbohydrate, g	209 ± 1	210 ± 2	211 ± 2	211 ± 2	0.61	238 ± 1	240 ± 3	246 ± 3*	245 ± 3	0.024
Starch, g	116 ± 1	113 ± 1	115 ± 2	109 ± 1*	0.0001	136 ± 1	135 ± 3	133 ± 2	134 ± 3	0.69
Total sugars, g	93.1 ± 1.3	97.3 ± 1.7	96.0 ± 1.8	103 ± 2*	0.0001	103 ± 1	104 ± 4	113 ± 3*	111 ± 3	0.004
IMS, g	33.1 ± 0.7	35.6 ± 1.0	37.4 ± 1.0*	43.2 ± 1.0*	0.0001	26.6 ± 0.5	30.9 ± 1.5*	33.8 ± 1.3*	41.8 ± 1.3*	0.0001
Fructose, g	16.1 ± 0.5	16.0 ± 0.6	16.5 ± 0.7	17.3 ± 0.6	0.42	16.9 ± 0.4	17.4 ± 1.1	19.0 ± 0.9	20.1 ± 0.9*	0.007
Lactose, g	11.9 ± 0.4	13.0 ± 0.5	13.8 ± 0.6*	16.1 ± 0.5*	0.0001	10.1 ± 0.3	11.1 ± 0.9	12.0 ± 0.8	13.3 ± 0.8*	0.001
Beta-carotene, μg	1940 ± 108	2050 ± 145	2110 ± 151	2170 ± 143	0.60	1870 ± 77	2190 ± 216	1970 ± 187	2950 ± 193*	0.0001
Vitamin A^b^, μg	614 ± 27	656 ± 36	698 ± 38	680 ± 36	0.25	634 ± 21	668 ± 59	660 ± 51	920 ± 53*	0.0001
Thiamin, mg	1.2 ± 0.0	1.3 ± 0.0	1.3 ± 0.0	1.3 ± 0.0*	0.005	1.4 ± 0.0	1.4 ± 0.0	1.5 ± 0.0	1.5 ± 0.0*	0.0001
Riboflavin, mg	1.4 ± 0.0	1.4 ± 0.0	1.5 ± 0.0*	1.7 ± 0.0*	0.0001	1.4 ± 0.0	1.5 ± 0.1	1.5 ± 0.1	1.7 ± 0.1*	0.0001
Folate, μg	193 ± 3	190 ± 5	204 ± 5	201 ± 4	0.07	204 ± 3	218 ± 8	223 ± 7*	248 ± 7*	0.0001
Vitamin B_12_, μg	3.7 ± 0.1	3.8 ± 0.1	4.1 ± 0.1	4.2 ± 0.1*	0.009	4.0 ± 0.1	4.3 ± 0.2	4.3 ± 0.2	4.4 ± 0.2	0.34
Vitamin D, μg	1.9 ± 0.1	2.0 ± 0.1	2.0 ± 0.1	2.0 ± 0.1	0.91	2.1 ± 0.0	2.1 ± 0.1	2.1 ± 0.1	2.5 ± 0.1*	0.03
Vitamin C, mg	83.5 ± 2.7	87.5 ± 3.6	81.5 ± 3.7	92.6 ± 3.5	0.11	76.5 ± 2.2	87.7 ± 6.2	92.9 ± 5	96.7 ± 5.6*	0.001
Sodium, mg	1837 ± 21	1849 ± 28	1917 ± 29	1770 ± 27	0.003	2268 ± 20	2249 ± 58*	2203 ± 50*	2123 ± 51	0.06
Potassium, mg	2098 ± 21	2104 ± 28	2147 ± 29	2289 ± 28*	0.0001	2248 ± 19	2356 ± 53	2407 ± 46*	2630 ± 48*	0.0001
Calcium, mg	739 ± 12	778 ± 16	827 ± 17*	901 ± 16*	0.0001	765 ± 10	820 ± 27	828 ± 24	856 ± 24*	0.001
Magnesium, mg	188 ± 2	185 ± 3	191 ± 3	203 ± 2*	0.0001	202 ± 2	218 ± 4*	216 ± 4*	240 ± 4*	0.0001
Phosphorus, mg	962 ± 9	975 ± 12	1013 ± 13*	1077 ± 12*	0.0001	1077 ± 8	1123 ± 23	1107 ± 20	1228 ± 21*	0.0001
Iron, mg	8.5 ± 0.1	8.6 ± 0.1	8.5 ± 0.2	8.8 ± 0.1	0.18	9.4 ± 0.1	10.0 ± 0.3	10.0 ± 0.2	10.5 ± 0.2*	0.0001
Zinc, mg	6.2 ± 0.1	6.3 ± 0.1	6.3 ± 0.1	6.7 ± 0.1*	0.007	7.3 ± 0.1	7.7 ± 0.2	7.4 ± 0.2	8.0 ± 0.2*	0.005
Iodine, μg	124 ± 3	132 ± 4	139 ± 4*	157 ± 4*	0.0001	119 ± 2	127 ± 7	136 ± 6*	152 ± 6*	0.0001
Selenium, μg	32.3 ± 0.5	31.6 ± 0.7	32.0 ± 0.8	34.67 ± 0.7*	0.009	39.6 ± 0.6	39.4 ± 1.6	40.8 ± 1.4	45.7 ± 1.4*	0.001
Manganese, mg	2.2 ± 0.0	2.1 ± 0.1	2.2 ± 0.1	2.2 ± 0.1	0.33	2.2 ± 0.0	2.5 ± 0.1*	2.4 ± 0.1	2.7 ± 0.1*	0.0001

Values shown are mean ± SEMs. Table contains nutrients that were significantly different across NC and increasing tertiles of yogurt and fromage frais intake

*IMS* intrinsic and milk sugars, *MUFAs* monounsaturated fatty acids, *NC* non-consumer, *NDNS* National Diet and Nutrition Survey, *SFAs* saturated fatty acids, *T* tertile

*Values are significantly different from non-consumers (*P* < 0.05; Bonferroni post hoc test)

^a^Based on differences across non-consumers and tertiles of yogurt intake by analysis of covariance (ANCOVA) controlling for age, sex and total energy intake (kJ). Energy intake was adjusted for age and sex

^b^Retinol equivalents

The percentage contribution of the children’s diets to recommended intakes for a number of nutrients were also higher for the highest tertile of yogurt intake (T3) compared with non-consumers (Supplemental Table 3). In children aged 4–10 years, the highest yogurt consumers (T3) had higher intakes of iodine (27%), calcium (25%), riboflavin (24%) and vitamin B_12_, (18%) compared with non-consumers. In children aged 11–18 years, the highest yogurt consumers (T3) had more vitamin A (34%), iodine (29%), vitamin C (26%), riboflavin (23%), folate (22%), potassium (22%), magnesium (22%), and selenium (22%) compared with non-consumers.

The percentage of children aged 4–10 years below the lower recommended nutrient intakes (LRNIs) was higher for non-consumers compared with children in the highest tertile of yogurt consumption (T3) for vitamins A, riboflavin, calcium, magnesium, iron, zinc, iodine and selenium (Table 3). The percentage of children aged 11–18 years below the lower recommended nutrient intakes (LRNIs) was higher for non-consumers compared with the diets of children in the highest tertile of yogurt intake (T3) for vitamins A, riboflavin, folate, vitamin B_12_, vitamin C, potassium, calcium, magnesium, iron, zinc, iodine and selenium.

**Table 3:** Percentage of British children aged 4–10 and 11–18 years from years 1–4 of the NDNS below the lower recommended nutrient intake (LRNIs) for non-consumers and across increasing tertile of yogurt and fromage frais intake

Component	Children 4–10 years Yogurt tertiles (g/day)				Children 11–18 years Yogurt tertiles (g/day)			
	NC (0)	T1 (1–30)	T2 (31–60)	T3 (61–295)	NC (0)	T1 (2–30)	T2 (31–60)	T3 (61–236)
Participants, *n*	307	166	155	175	610	97	89	88
Vitamin A^a^	8.8	3.6	7.1	5.7	15.7	6.5	7.9	5.1
Riboflavin	1.3	0.6	1.3	0.0	19.0	10.4	8.9	4.1
Vitamin B_6_	0.0	0.0	0.0	0.0	0.2	0.0	0.0	0.0
Folate	0.0	0.0	0.0	0.0	6.2	1.3	2.0	1.0
Vitamin B_12_	0.0	0.6	0.6	0.0	2.3	0.0	0.0	0.0
Vitamin C	0.3	0.0	0.0	0.0	1.3	0.0	0.0	0.0
Potassium	0.3	0.0	0.0	0.0	28.9	13.0	15.8	6.1
Calcium	2.9	2.4	0.6	0.0	16.6	10.4	4.0	3.1
Magnesium	2.9	3.0	0.6	0.0	45.7	24.7	32.7	17.3
Iron	2.0	2.4	1.3	1.1	29.0	18.2	20.8	16.3
Zinc	11.7	9.6	7.7	3.4	16.6	13.0	19.8	7.1
Iodine	6.8	3.0	2.6	0.0	19.3	13.0	5.9	1.0
Selenium	1.3	1.2	1.3	0.0	38.4	16.9	23.8	15.3

Values are percentages. There were no children below the LRNI for thiamin and niacin, so this data is not shown in the table

*LRNI* Lower recommended nutrient intake, *NC* non-consumer, *NDNS* National Diet and Nutrition Survey, *T* tertile

^a^Retinol equivalents

### Intakes of other food groups and overall diet quality

The consumption of different food groups varied significantly between non-consumers and across tertiles of yogurt intake (Table 4). In children aged 4–10 years, higher yogurt consumption (T3) was associated with significantly (*P* < 0.05) lower intakes of processed meat and buns, cakes and pastries, and significantly (*P* < 0.05) higher intakes of biscuits and high fibre breakfast cereals compared to non-consumers. In children aged 11–18 years, higher yogurt consumption (T3) was associated with significantly less (*P* < 0.05) processed meat and white bread, and significantly (*P* < 0.05) more high fibre bread, high fibre breakfast cereals, other dairy foods, fish and fish dishes, fruit and vegetables, fruit juice, tea coffee and water and nuts and seeds.

**Table 4:** Intakes of other food groups and HEI-2010 score for children aged 4–10 and 11–18 years for non-consumers and across increasing tertile of yogurt and fromage frais intake using data from years 1–4 of the NDNS

Food groups, g/d	Children 4–10 years Yogurt tertiles (g/day)				*P* ^2^	Children 11–18 years Yogurt tertiles (g/day)				*P*
	NC (0)	T1 (1–30)	T2 (31–60)	T3 (61–295)		NC (0)	T1 (2–30)	T2 (31–60)	T3 (61–236)	
Participants, *n*	307	166	155	175		610	97	89	88	
Total HEI-2010 score (out of 100)	57.3 ± 0.5	57.6 ± 0.7	58.9 ± 0.7	62.9 ± 0.6*	0.0001	55.4 ± 0.3	58.6 ± 1.0*	60.1 ± 0.8*	66.3 ± 0.9*	0.0001
Pasta, rice and other cereals	71.3 ± 3.2	71.7 ± 4.4	65.8 ± 4.5	62.0 ± 4.3	0.28	102 ± 3	106 ± 10	91.9 ± 8.3	90.9 ± 8.5	0.43
White bread	42.5 ± 2.1	45.2 ± 2.8	50.0 ± 2.9	41.0 ± 2.7	0.12	60.1 ± 1.8	54.7 ± 5.0	57.7 ± 4.3	46.8 ± 4.5*	0.048
Bread (high fibre)	22.1 ± 1.8	19.9 ± 2.4	24.9 ± 2.5	25.0 ± 2.4	0.37	17.0 ± 1.2	21.2 ± 3.5	21.0 ± 3.0	29.8 ± 3.1*	0.002
Other bread	2.2 ± 0.5	2.0 ± 0.7	2.2 ± 0.7	2.5 ± 0.7	0.97	1.6 ± 0.4	2.6 ± 1.1	3.0 ± 1.0	3.3 ± 1.0	0.27
Breakfast cereals (high fibre)	17.8 ± 1.6	13.1 ± 2.1	15.6 ± 2.2	22.1 ± 2.1*	0.018	10.6 ± 0.9	15.8 ± 2.7	11.2 ± 2.3	17.0 ± 2.4*	0.042
Other breakfast cereals	9.8 ± 0.7	10.7 ± 1.0	10.0 ± 1.0	9.7 ± 0.9	0.85	10.3 ± 0.7	9.4 ± 1.9	11.3 ± 1.7	11.2 ± 1.7	0.85
Biscuits	14.1 ± 0.9	17.4 ± 1.2	18.8 ± 1.3*	16.6 ± 1.2*	0.016	15.8 ± 0.9	15.3 ± 2.6	20.2 ± 2.3	16.6 ± 2.4	0.34
Buns, cakes, pastries	39.7 ± 2.0	36.4 ± 2.7	32.6 ± 2.8	29.6 ± 2.7*	0.018	28.8 ± 1.6	28.4 ± 4.5	30.8 ± 3.9	40.5 ± 4.0	0.06
Milk	184 ± 9	195 ± 12	204 ± 12	218 ± 11	0.12	144 ± 7	151 ± 20	141 ± 17	129 ± 18	0.85
Cheese	9.0 ± 0.7	9.6 ± 0.9	11.3 ± 1.0	10.7 ± 0.9	0.22	11.3 ± 0.6	10.9 ± 1.6	10.9 ± 1.4	11.1 ± 1.5	0.99
Yogurt and fromage frais	0.0 ± 0.0	19.4 ± 7.8*	43.2 ± 8.3*	98.4 ± 35.7*	0.0001	0.0 ± 0.0	19.6 ± 1.5*	40.9 ± 1.3*	105 ± 1*	0.0001
Other dairy foods	53.9 ± 4.7	45.4 ± 6.4	45.0 ± 6.6	43.1 ± 6.2	0.47	21.2 ± 2.1	22.5 ± 5.9	40.5 ± 5.1*	22.5 ± 5.3*	0.006
Eggs and dishes	10.3 ± 1.0	12.0 ± 1.4	7.4 ± 1.4	8.7 ± 1.4	0.10	11.8 ± 0.9	13.8 ± 2.6	10.3 ± 2.2	16.5 ± 2.3	0.19
Fat spreads and oils	9.0 ± 0.4	7.8 ± 0.5	10.0 ± 0.6	8.2 ± 0.5	0.024	8.6 ± 0.3	7.2 ± 0.9	8.1 ± 0.8	8.2 ± 0.8	0.54
Red meat	30.4 ± 2.5	31.6 ± 3.4	32.5 ± 3.5	35.0 ± 3.3	0.76	49.0 ± 2.6	57.5 ± 7.4	51.5 ± 6.4	49.5 ± 6.6	0.747
White meat	38.2 ± 2.3	35.6 ± 3.2	35.4 ± 3.3	31.6 ± 3.1	0.41	65.5 ± 2.5	58.2 ± 7.1	54.7 ± 6.2	55.6 ± 6.4	0.23
Processed meat	39.6 ± 1.8	34.1 ± 2.5	34.0 ± 2.5	31.1 ± 2.4*	0.030	52.1 ± 2.0	51.7 ± 5.7	49.6 ± 4.9	33.4 ± 5.0*	0.008
Other meat and offal	1.9 ± 0.5	2.9 ± 0.7	2.7 ± 0.7	2.1 ± 0.7	0.67	3.5 ± 0.6	2.5 ± 1.6	3.0 ± 1.4	1.4 ± 1.4	0.56
Fish and fish dishes	20.9 ± 1.4	15.9 ± 1.9	15.3 ± 2.0	21.6 ± 1.9	0.021	16.3 ± 1.2	16.3 ± 3.5	19.5 ± 3.0	26.3 ± 3.1*	0.024
Fruit and vegetables	240 ± 7	243 ± 9	236 ± 9	254 ± 9	0.55	210 ± 5	227 ± 14	229 ± 12	297 ± 12*	0.0001
Nuts and seeds	0.7 ± 0.2	1.0 ± 0.3	0.8 ± 0.3	1.1 ± 0.3	0.74	1.0 ± 0.2	1.9 ± 0.6	1.2 ± 0.5	2.6 ± 0.5*	0.024
Savoury snacks	10.3 ± 0.6	9.9 ± 0.7	11.3 ± 0.8	8.4 ± 0.7	0.05	13.7 ± 0.6	12.7 ± 1.7	14.4 ± 1.5	11.7 ± 1.5	0.55
Sugars, preserves	5.5 ± 0.4	5.2 ± 0.6	5.6 ± 0.6	4.9 ± 0.6	0.79	6.5 ± 0.4	7.1 ± 1.1	4.9 ± 1.0	5.8 ± 1.0	0.37
Confectionery	18.0 ± 1.2	19.8 ± 1.7	17.1 ± 1.7	14.2 ± 1.6	0.11	20.4 ± 0.9	21.6 ± 2.6	18.2 ± 2.3	15.5 ± 2.4	0.20
Fruit juice	97.6 ± 7.0	88.0 ± 9.4	85.5 ± 9.8	90.8 ± 9.3	0.74	71.8 ± 5.0	95.5 ± 14.2	108 ± 12*	107 ± 13*	0.005
Soft drinks	312 ± 15	313 ± 21	324 ± 21	303 ± 21	0.92	470 ± 14	432 ± 41	426 ± 35	374 ± 36	0.08
Tea, coffee and water	286 ± 14	250 ± 19	235 ± 20	280 ± 19	0.13	414 ± 17	588 ± 49	436 ± 43	484 ± 44*	0.008
Alcohol	0.0 ± 0.0	0.0 ± 0.0	0.0 ± 0.0	0.0 ± 0.0	1.000	42.3 ± 9.2	63.9 ± 26.1	66.7 ± 22.6	40.1 ± 23.3	0.68
Dry weight beverages	2.0 ± 0.5	2.5 ± 0.7	2.1 ± 0.7	3.0 ± 0.7	0.66	2.1 ± 0.4	2.1 ± 1.2	2.3 ± 1.1	2.8 ± 1.1	0.95
Condiments	14.7 ± 1.0	16.9 ± 1.4	14.7 ± 1.5	13.7 ± 1.4	0.41	22.9 ± 1.1	26.4 ± 3.1	21.0 ± 2.6	23.5 ± 2.7	0.62
Soups	10.6 ± 2.0	14.7 ± 2.7	16.9 ± 2.8	13.2 ± 2.6	0.29	15.0 ± 1.8	15.0 ± 5.2	12.7 ± 4.5	17.2 ± 4.6	0.92

Values shown are mean ± SEMs after adjustment

*HEI-2010* healthy eating index-2010, *NC* non-consumer, *NDNS* National Diet and Nutrition Survey, *T* tertile

*Values are significantly different from non-consumers (*P* < 0.05; Bonferroni post hoc test)

^a^Based on differences across non-consumers and tertiles of yogurt intake by ANCOVA controlling for age, sex and total energy intake (kJ)

The HEI-2010 was significantly higher in the high yogurt tertile (T3) compared with non-consumers for children aged 4–10 and 11–18 years (both *P* = 0.0001) (Table 4). Furthermore, the adjusted component scores for total fruit, whole fruit and dairy portions for children aged 4–10 years were significantly higher (*P* = 0.022, *P* = 0.004 and *P* = 0.0001, respectively) for the high yogurt consumers (T3) compared with non-consumers (Supplemental Table 4). Fatty acids (ratio of mono- and poly-unsaturated fatty acids to saturated fat) were significantly lower (*P* = 0.0001) in children in the highest tertile (T3) of yogurt intake compared to non-consumers. Refined grains were significantly lower (*P* = 0.024) in children in the lowest quartile (T1) of yogurt intake compared with non-consumers. For children aged 11–18 years, the adjusted component scores for total fruit, whole fruit, green beans, wholegrain, dairy portions and seafood and plant protein was significantly (all *P* = 0.0001) higher for the highest tertile (T3) of yogurt consumers compared with non-consumers. Fatty acid intake was significantly lower (*P* = 0.024) in the highest tertile of yogurt intake (T3) compared with non-consumers.

## Metabolic profiles

The metabolic profiles of children aged 4–10 years were significantly different for some parameters for yogurt consumers compared with non-consumers (Table 5). For adjusted values please see Supplemental Table 5. When controlling for total energy intake, BMI, age and sex (model 2), there was a significant difference in pulse pressure (*P* = 0.024) across non-consumers and tertiles of yogurt intake, with children in the highest tertile (T3) of yogurt consumption having lower pulse pressure compared with non-consumers. This result remained significant (*P* = 0.038) after further adjustment for HEI-2010 score (model 3). There were no other significant differences in metabolic parameters in children aged 4–10 years.

**Table 5:** Metabolic profiles of children aged 4–10 and 11–18 years for non-consumers and across increasing tertile of yogurt and fromage frais intake using data from years 1–4 of the NDNS

Biomarkers	Children 4–10 years Yogurt tertiles (g/day)						
	NC (0)	T1 (1–30)	T2 (31–60)	T3 (61–295)	*P* values^a^		
					Model 1^b^	Model 2^c^	Model 3^d^
Participants, *n*	307	166	155	175			
Height, cm	125.6 ± 0.8	124 ± 1.1	122.8 ± 1.1	126.4 ± 1.0	0.07	0.71	0.83
Weight, kg	27.8 ± 0.5	26.9 ± 0.7	25.9 ± 0.7	28.2 ± 0.7	0.07	0.88	0.90
BMI, kg/m^2^	17.2 ± 0.2	17.1 ± 0.2	16.8 ± 0.2	17.4 ± 0.2	0.34	0.59	0.62
SBP, mm Hg	103.9 ± 0.7	103.5 ± 0.9	103.1 ± 0.9	105 ± 0.8	0.48	0.80	0.52
DBP, mm Hg	62.9 ± 0.6	63.7 ± 0.8	62.0 ± 0.9	62.9 ± 0.8	0.62	0.46	0.53
PP, mm Hg	87.2 ± 0.8	85.9 ± 1.0	85.4 ± 1.1	84.4 ± 1.0	0.14	0.024	0.038
Serum cholesterol, mmol/L	4.5 ± 0.1	4.3 ± 0.1	4.2 ± 0.1	4.5 ± 0.1	0.23	0.16	0.16
Serum HDL-C, mmol/L	1.6 ± 0.1	1.5 ± 0.1	1.7 ± 0.1	1.6 ± 0.6	0.43	0.60	0.61
Serum LDL-C, mmol/L	2.6 ± 0.1	2.5 ± 0.1	2.4 ± 0.1	2.6 ± 0.1	0.35	0.21	0.22
HDL ratio	2.9 ± 0.1	3.0 ± 0.1	2.6 ± 0.1	2.9 ± 0.1	0.15	0.23	0.23
Serum TAG, mmol/L	0.8 ± 0.1	0.7 ± 0.1	0.6 ± 0.1	0.7 ± 0.6	0.24	0.37	0.37
Serum CRP, mg/L	1.6 ± 0.3	1.5 ± 0.4	2.4 ± 0.4	1.6 ± 0.3	0.25	0.15	0.15
Plasma HbA1c, %	5.3 ± 0.04	5.3 ± 0.1	5.3 ± 0.1	5.3 ± 0.1	0.94	0.85	0.84
Plasma glucose, mmol/L	4.8 ± 0.1	4.7 ± 0.1	4.7 ± 0.1	4.7 ± 0.1	0.77	0.63	0.48

Biomarkers	Children 11–18 years Yogurt tertiles (g/day)						
	NC (0)	T1 (2–30)	T2 (31–60)	T3 (61–236)	*P* values		
					Model 1	Model 2	Model 3
Participants, *n*	610	97	89	88			
Height, cm	164.6 ± 10.8	160.9 ± 10.2	164.0 ± 11.6	161.4 ± 12.6	0.005	0.033	0.035
Weight, kg	60.2 ± 15.3	57.3 ± 16.9	58.6 ± 15.3	56.9 ± 16.1	0.13	0.09	0.09
BMI, kg/m^2^	22.0 ± 4.4	21.9 ± 5.3	21.6 ± 4.3	21.5 ± 4.2	0.59	0.95	0.84
Waist, cm	76.9 ± 11.1	74.1 ± 11.8	75.5 ± 11.2	75.0 ± 10.7	0.21	0.18	0.28
Hip, cm	94.8 ± 10.2	92.0 ± 11.0	94.1 ± 9.6*	92.3 ± 10.8	0.10	0.009	0.008
Waist:hip ratio	0.8 ± 0.1	0.8 ± 0.1	0.8 ± 0.1	0.8 ± 0.1	0.54	0.37	0.67
SBP, mm Hg	113.4 ± 11.3	109.7 ± 9.7	112.5 ± 10.6	114.8 ± 10.3	0.07	0.20	0.13
DBP, mm Hg	63.6 ± 8.5	63.2 ± 7.6	63.7 ± 8.7	63.7 ± 7.7	0.98	0.99	0.99
PP, mm Hg	74.3 ± 10.6	76.1 ± 11.6	74.9 ± 11.0	73.5 ± 9.7	0.58	0.39	0.70
Serum cholesterol, mmol/L	4.1 ± 0.7	4.1 ± 0.8	3.9 ± 0.7	3.9 ± 0.7	0.23	0.19	0.15
Serum HDL-C, mmol/L	1.4 ± 0.3	1.4 ± 0.2	1.4 ± 0.2	1.4 ± 0.4	0.93	0.99	0.99
Serum LDL-C, mmol/L	2.3 ± 0.6	2.4 ± 0.7	2.1 ± 0.6	2.2 ± 0.5	0.23	0.21	0.18
HDL ratio	3.0 ± 0.8	2.9 ± 0.7	2.7 ± 0.6	2.8 ± 0.6	0.17	0.30	0.41
Serum TAG, mmol/L	0.9 ± 0.4	0.8 ± 0.5	0.8 ± 0.3	0.8 ± 0.5	0.52	0.82	0.98
Serum CRP, mg/L	1.8 ± 0.8	1.7 ± 0.2	1.5 ± 0.2	1.7 ± 0.2	0.72	0.68	0.60
Plasma HbA1c, %	5.3 ± 0.3	5.3 ± 0.3	5.3 ± 0.3	5.1 ± 0.3*	0.026	0.005	0.010
Plasma glucose, mmol/L	4.8 ± 0.4	4.8 ± 0.3	4.8 ± 0.3	4.7 ± 0.4	0.55	0.26	0.45

Values are mean ± SEMs For children aged 4–10 years there were no data on waist, hip and waist:hip ratio

*CRP* C-reactive protein, *DBP* diastolic blood pressure, *HbA1c* glycated haemoglobin, HDL-C, *NDNS* national diet and nutrition survey, *NC* non-consumer, *N/A* not available, *PP* pulse pressure, *SBP* systolic blood pressure, *TAGs* Triacylglycerols, *T* tertile

*Values are significantly different from non-consumers in model 3 (*P* < 0.05; Bonferroni post hoc test)

^a^Based on differences across non-consumers and tertiles of yogurt intake by ANCOVA

^b^Non-adjusted

^c^Adjusted for age, sex, BMI and total energy intake (kJ/d)

^d^Additional adjustment for HEI-2010 score

For children aged 11–18 years, height, hip circumference and HbA1c concentrations were significantly different across tertiles of yogurt consumption when controlling for age, sex, BMI and total energy intake (model 2), with the highest tertile of yogurt intake (T3) being the shortest and having the smallest hip circumference as well as the lowest HbA1c concentrations compared with non-consumers. When controlling for age, sex, BMI, total energy intake and HEI-2010 score (model 3) the differences in height, hip circumference and HbA1c across increasing tertiles of yogurt intake remained significantly different (*P* = 0.035, *P* = 0.008 and *P* = 0.010, respectively). There were no significant other differences in metabolic profiles of children aged 4–18 years across increasing tertiles of yogurt consumption.

### Dietary under-reporting

In children aged 4–10 years, 15%, 77% and 8% were identified as under-reporters, feasible reporters and over-reporters, respectively and the exclusion of under-reporters in the statistical analysis did not result in any significant differences in mean nutrient intakes or metabolic profile. In children aged 11–18 years, 47%, 50% and 3% were identified as under-reporters, feasible reporters and over-reporters, respectively. Following the exclusion of subjects identified as under-reporters intakes of thiamin, sodium and total sugars became non-significant across tertiles of yogurt intake. There were no other significant differences across yogurt tertiles in nutrient intakes or metabolic profile.

## Discussion

This study was the first to investigate the association between yogurt consumption and nutritional intakes and adequacy, diet quality and metabolic profiles in children using data from years 1–4 of the NDNS, UK. We found that 62% of children aged 4–10 years and 31% of children aged 11–18 years were yogurt consumers (> 0 g yogurt/d). The highest tertile (T3) of yogurt consumption was associated with higher nutritional intakes and adequacy, as well as better diet quality (assessed by HEI-2010) compared with non-yogurt consumers for both age groups. Yogurt consumption was associated with significantly lower pulse pressure in children aged 4–10 years and lower HbA1c concentrations, height and hip circumference in children aged 11–18 years, compared with non-yogurt consumers.

The finding that yogurt consumption is higher in younger children (4–10 years) compared with older children (11–18 years) is similar to studies conducted in other populations. For example, Zhu and colleagues (2015) found that around 72% of American children aged 2–11 consumed yogurt ≥ 1 times a week, compared with 28% of children aged 12–18 years [14]. One explanation for this may be that teenagers are more independent in their food choices whereas parents would be more in control of food choices for younger children. Dietary surveys have highlighted that teenage girls often have intakes below the lower recommended nutrient intake (LRNI) for a number of key nutrients such as calcium, magnesium, potassium, zinc, selenium and iodine. These nutrients are found in yogurt and other dairy products and therefore higher intakes in this population may be of benefit [1].

Children in the highest tertile (T3) of yogurt consumption for both age groups also had higher intakes, as well as fewer children below the LRNI, for a number of nutrients, particularly vitamin A, riboflavin, vitamin B_12_, vitamin C, potassium, calcium, zinc and iodine. This is in line with the findings of other studies, which have also found that dairy and/or yogurt consumption was associated with improved nutrient intakes and adequacy in adults [30–33] and children [12, 14]. Given that yogurt is a good source of a number of these nutrients it is likely that the higher yogurt intakes of the children in the high yogurt tertile (T3) contributed towards the increased nutrient intakes and adequacy, although this requires confirmation in further studies. However, the higher nutrient intakes and adequacy may also be because these children had a better overall diet quality (assessed by HEI-2010) compared to non-yogurt consumers, which reflected their overall dietary patterns. For example, the diets of children aged 4–10 years consuming the highest amount of yogurt were associated with lower intakes of fat spread and oils, processed meat and buns, cakes, pastries, puddings and pies, and higher intakes fish and fish dishes, biscuits and high fibre breakfast cereals. The diets of children aged 11–18 years consuming the highest amount of yogurt were associated with less processed meat and white bread, and more high fibre bread, high fibre cereals, fruit and vegetables, fish, fruit juice, tea, coffee and water and nuts and seeds. In their study of American children Zhu and colleagues (2015) also found that diet quality, assessed using HEI-2005, was higher in children consuming yogurt compared with non-consumers [14]. In addition, several HEI-2005 component scores were also higher in the yogurt consumers, suggesting a better overall compliance to nutrient recommendations. In our study, we used the HEI-2010, which is based on 2010 dietary guidelines for Americans, however where possible UK dietary guidelines were used as this would be better suited for use with UK data. Although there are some differences in the definition of food groups between the HEI-2005 and HEI-2010, it has been proposed by Guenther et al. that this would be unlikely to greatly affected comparison of studies using these 2 HEI scores [34].

We found that yogurt consumption was associated with lower pulse pressure in children aged 4–10 years and lower hip circumference, height and HbA1c in children aged 11–18 years, which when controlling for HEI-2010 score remained significantly different. This suggests that the effects observed are more strongly associated with yogurt intake rather than other components of the diet.

In this study we did not find any differences in lipid profile in British children, which is supported by the majority of other studies in adults [35] and children [14]. For example, Van der Water and colleagues (1999) showed that consumption of either 200 g live culture or pasteurized yogurt per day for one year resulted in stable LDL-C concentrations, however in the control group (no yogurt) total cholesterol and LDL-C concentrations were significantly higher after the study duration [35]. Saturated fatty acid intake has been associated with elevated total-C and LDL-C concentrations. In our study, we did not find a significant difference in saturated fatty acids across yogurt tertiles, which may also be a possible explanation for why we did not find any differences in lipid profile in children with varying levels of yogurt intake.

We did not find any differences in waist hip ratio, systolic blood pressure, diastolic blood pressure or glucose concentrations between yogurt consumers and non-consumers or with increasing tertiles of yogurt intake, which is supported by findings from another study in children [14]. Furthermore, despite significantly higher total energy and sugar intake in children consuming the highest amount of yogurt (T3) there were no significant differences in bodyweight or BMI. This could also have been as a result of increased physical activity in this group, however we were not able to control for this, as this data was very limited for our populations. However, a recent systematic review of 6 cohort and 7 cross-sectional studies found that yogurt consumption was associated with lower BMI, bodyweight/weight gain, smaller waist circumference and lower body fat levels in adults [36].

The finding that HbA1c concentration was significantly lower in the highest tertile of yogurt consumers (T3) compared with non-consumers for children aged 11–18 years is in line with previous studies. Insulin concentrations were not determined, but other studies have shown yogurt consumption to be associated with lower fasting insulin concentrations in American children [14] and adults [37]. There is also evidence to suggest that fermented milk and yogurt products may have potential antihypertensive effects, which has been attributed, in part, to the presence of lactotripeptides in these products [38]. A number of randomized control trials and meta-analyses have shown that some tripeptides derived from certain milk protein, lower blood pressure by inhibition of angiotensin 1 converting enzyme, production of vasodilators, or an effect on nervous activity [39, 40]. This could be a possible explanation for the lower pulse pressure observed for children aged 4–10 years in the highest tertile (T3) of yogurt intake compared with non-consumers, although this requires confirmation.

Our analysis of energy misreporting indicated that 15% of children aged 4–10 years were classed as under-reporters, but the exclusion of these subjects in the sensitivity statistical analysis did not result in significant differences in nutrient intakes or metabolic profile across yogurt tertiles. The number of subjects classed as under-reporters were much higher (47%) for children aged 11–18 years, and following their exclusion in the sensitivity analysis, significant differences were lost for thiamin, sodium and total sugars. The reasons for this are unclear, but could reflect a lower statistical power (47% reduction in sample size). The rates of misreporting in our study are similar to those reported in other studies in children using the same methodology [22, 27]. The increasing bias towards under-reporting as children get older has often been described [41, 42] and a number of reasons have been suggested such as eating behaviours and food patterns being less structures in adolescence [43], increased eating away from the home, particularly snacks, which are more likely to be forgotten. In addition, children in the 11–18-years-old age group are more likely to complete their own diet diary, whereas in the younger age group (4–10 years) dietary intake would usually be reported by a proxy person. The children completing their own food diary may therefore have been more tempted to report food intakes in line with dietary guidelines, particularly if they are overweight [44].

Another limitation of the current study is the calculation of HEI-2010, which is developed using American dietary guidelines, because the UK does not currently have its own HEI score. Therefore, our calculation of the HEI-2010 was estimated using the UK dietary guidelines, which limits direct comparisons with our HEI-2010 scores and those calculated in other studies. Furthermore, because one of the HEI-2010 component scores was dairy, which included yogurt intake, it is possible that the overall HEI-2010 score across increasing tertiles of yogurt intake for our population would also be higher. However, other component scores such as total fruit, whole fruit, whole-grains and green beans were also significantly different across increasing yogurt tertiles suggesting that it is not just the dairy component driving the HEI-2010 score. Another potential limitation is the cross-sectional design of the NDNS, where observed associations cannot imply causation. In addition, dietary assessment in children and adolescents is complicated by a variety of factors, including reliance on a third person (e.g., parent, adult caregiver or teacher) to report a child’s intake during early years, cognitive abilities of the child, motivation and reporting biases (e.g., under- or over-reporting), all of which are influenced by developmental stage of the child. In our statistical analysis we did not adjust for multiple testing and due to the many comparisons that were done in each analysis, the *p* values obtained in this study, particularly for differences across yogurt tertiles and metabolic profile should be interpreted with caution. Furthermore, there may be a differential effect of yogurt types on cardio-metabolic risk factors. However, this study did not have sufficient power to detect differences between yogurt types.

## Conclusion

This study found that dietary patterns that included yogurt were associated with higher intakes of certain beneficial nutrient and overall better diet quality in children (4–18 years). Furthermore, dietary patterns that included yogurt were associated with lower pulse pressure and HbA1c concentrations in children aged 4–10 and 11–18 years, respectively, although no association was found between other metabolic outcomes measured. Additional long-term randomized controlled trials are needed to further examine the effects of yogurt consumption on cardio-metabolic health and to establish if yogurt is the key driver of these in children. In summary, including yogurt as part of children’s diets may be a strategy for increasing intakes of certain nutrients particularly calcium, magnesium, iodine and riboflavin, although this would need confirmation in further studies.

## Electronic supplementary material

Below is the link to the electronic supplementary material.

Supplementary material 1 (DOCX 97 KB)

Supplementary material 2 (DOCX 90 KB)

Supplementary material 3 (DOCX 121 KB)

Supplementary material 4 (DOCX 104 KB)

Supplementary material 5 (DOCX 119 KB)

Supplementary material 6 (DOCX 26 KB)

## Abbreviations

ANOVA: Analysis of variance
BMI: Body mass index
CVD: Cardiovascular disease
CHD: Coronary heart disease
HEI: Healthy eating index
HDL-cholesterol: High-density lipoprotein cholesterol
HbA1c: Glycated haemoglobin
LRNI: Lower recommended nutrient intake
LDL-cholesterol: Low-density lipoprotein cholesterol
MUFA: Monounsaturated fatty acids
NDNS: National diet and nutrition survey
NHANES: National Health and Nutrition Examination Survey
TAG: Triacylglycerol
