# Supplementary material for: Yogurt consumption is associated with higher nutrient intake, diet quality and favourable metabolic profile in children: a cross-sectional analysis using data from years 1–4 of the National diet and Nutrition Survey, UK
Source: Eur J Nutr. 2018 Jan 12;58(1):409–22. doi: 10.1007/s00394-017-1605-x (PMC6424923; doi:10.1007/s00394-017-1605-x)
Supplement: Supplementary file 1 — Supplementary material 1 (DOCX 97 KB) [file 394_2017_1605_MOESM1_ESM.docx]

**Supplemental Methods 1.** Calculation of Healthy Eating Index-2010

Diet quality was measured using the Healthy Eating Index 2010 (HEI-2010) with some modifications, which were based on the 2007 dietary guidelines for UK institutions ([20](#_ENREF_20)). (**Supplemental Methods 1** and **Supplemental Table 1**). It was decided to use HEI because it is the most suited indicator of diet quality of children’s diets and has been used previously in this population ([18](#_ENREF_18)). A description of the methodology has been described in detail previously ([21](#_ENREF_21)) and steps for calculating HEI-2010 are also available from the National Cancer Institute ([36](#_ENREF_36)) and the National Institute on Ageing ([37](#_ENREF_37)). Briefly, the HEI-2010 scores 12 food groups/components for a total of 100 points, with a maximum score being achieved if diets meet recommendations for a particular food group/component, and a lower score assigned if diets do not meet recommendations. Six of the components (total fruit, whole fruit, total vegetables, greens and beans, seafood and plant proteins, and total protein foods) were worth 0-5 points, 5 components (whole-grains, dairy [included milks, cheese, yogurt, fromage frais, dairy desserts], fatty acids ratio [(PUFA + MUFA/SF)], refined grains and sodium) were worth 0-10 points, and one component (empty calories, which included energy from solid fats, added sugars) was worth 0-20 points and is reverse scored, such as a lower intake was given a better score. Refined grains and sodium were also reverse scored. The same dietary guidelines were applied to both age groups, apart from for sodium, which was scored according to age (4-6 y, 7-10 y and 11+ y), because UK sodium guidelines are different for these age groups. All components except the fatty acids ratio were scored on a density basis (per 1,000 kcal) or as a percentage of total energy. **Supplemental Table 1** shows the HEI-2010 components, food group modifications, standards for scoring and if the scores are based on US or UK dietary guidelines.
