# Supplementary material for: Yogurt consumption is associated with higher nutrient intake, diet quality and favourable metabolic profile in children: a cross-sectional analysis using data from years 1–4 of the National diet and Nutrition Survey, UK
Source: Eur J Nutr. 2018 Jan 12;58(1):409–22. doi: 10.1007/s00394-017-1605-x (PMC6424923; doi:10.1007/s00394-017-1605-x)
Supplement: Supplementary file 2 — Supplementary material 2 (DOCX 90 KB) [file 394_2017_1605_MOESM2_ESM.docx]

**Supplemental Table 1** Healthy Eating Index-2010 components and standards for scoring^1^

| Component | Modifications | Scoring points/scale | | Standards for points | | Dietary  Guidelines  /recommendations |
| --- | --- | --- | --- | --- | --- | --- |
|  |  | Max | Min | Max | Min |  |
| Adequacy |  |  |  |  |  |  |
| Total fruit^2^, g | All fruit and fruit juice incl composite dishes | 5 | 0 | ≥160 | 0 | UK |
| Whole fruit, g | All whole fruits, canned, frozen, dried | 5 | 0 | ≥80 | 0 | UK |
| Total vegetables, g^3^ | All vegetables incl composite dishes | 5 | 0 | ≥240 | 0 | UK |
| Green and beans, g | Yellow, red, green vegetables, beans, peas | 5 | 0 | ≥45 | 0 | US |
| Whole grains, g | Wholegrain/high fiber breakfast cereals, wholemeal breads and crackers | 10 | 0 | ≥42 | 0 | US |
| Dairy portions^4^ | Milks, yogurt and fromage frais, cheese | 10 | 0 | 3 | 0 | UK |
| Total protein foods, g | Beef, veal, lamb, pork | 5 | 0 | ≥70 | 0 | US |
| Seafood and plant proteins, g | Seafood, nuts and seeds | 5 | 0 | ≥22 | 0 | US |
| Fatty acids | Ratio of PUFAs+MUFAs to SFAs | 10 | 0 | ≥2.5 | ≤1.2 | US |
| Moderation |  |  |  |  |  |  |
| Refined grains, g | Biscuits, white bread, breakfast cereals not high in fiber, white pasta, white rice and other cereal products | 5 | 0 | ≤50 | ≥120 | US |
| Sodium, g | Calculated for three age groups:  4-6 y  7-10 y  >11y | 10  10  10 | 0  0  0 | ≤0.6  ≤1.1  ≤1.2 | ≥1.2  ≥2  ≥2.4 | UK |
| Empty calories, % | NMES and alcohol (% total energy intake) did not include solid fats | 20 | 0 | ≤34.5 | ≥50 | US |

^1^ All weight conversions are based on 1 oz and 1 cup (American measures) being equivalent to 28 g and 225 g, respectively. PUFAs, polyunsaturated fatty acids; MUFAs, monounsaturated fatty acids; SFAs, saturated fatty acids; NMES, non-milk extrinsic sugars.

^2^ Based on 2 x 80 g portions of fruit per day.

^3^ Based on 3 x 80 g portions of vegetables per day.

^4^ Based on portions of dairy consumed per day with 200 mL of milk, 150 mL yogurt and 30 g cheese being equivalent to 1 portion of dairy.
