# Supplementary material for: Yogurt consumption is associated with higher nutrient intake, diet quality and favourable metabolic profile in children: a cross-sectional analysis using data from years 1–4 of the National diet and Nutrition Survey, UK
Source: Eur J Nutr. 2018 Jan 12;58(1):409–22. doi: 10.1007/s00394-017-1605-x (PMC6424923; doi:10.1007/s00394-017-1605-x)
Supplement: Supplementary file 3 — Supplementary material 3 (DOCX 121 KB) [file 394_2017_1605_MOESM3_ESM.docx]

**SUPPLEMENTAL TABLE 2** Nutritional adequacy for the non-yogurt consumers and across increasing tertile of yogurt and fromage frais consumption using data from years 1-4 of the NDNS for children aged 4-10 and 11-18 years old^1^

|  | Children 4-10 y  Yogurt tertiles (g/d) | | | |  |  | Children 11-18 y  Yogurt tertiles (g/d) | | | |  |
| --- | --- | --- | --- | --- | --- | --- | --- | --- | --- | --- | --- |
| Nutrients per day | NC  (0) | T1  (1-30) | T2  (31-60) | T3  (61-295) | *P*^2^ |  | NC  (0) | T1  (2-30) | T2  (31-60) | T3  (61-236) | *P* |
| Participants, n | 307 | 166 | 155 | 175 |  |  | 610 | 97 | 89 | 88 |  |
| SFA, g | 22.4 ± 0.3 | 22.7 ± 0.3 | 22.5 ± 0.4 | 22.4 ± 0.3 | 0.94 |  | 25.2 ± 0.2 | 25.3 ± 0.6 | 24.1 ± 0.5* | 24.5 ± 0.5* | 0.16 |
| n-3 fatty acids, g | 1.4 ± 0.0 | 1.4 ± 0.0 | 1.3 ± 0.0 | 1.3 ± 0.0 | 0.05 |  | 1.8 ± 0.0 | 1.7 ± 0.1 | 1.7 ± 0.1 | 1.7 ± 0.1 | 0.12 |
| Trans fatty acids, g | 1.1 ± 0.0 | 1.1 ± 0.0 | 1.0 ± 0.0 | 1.1 ± 0.0 | 0.74 |  | 1.2 ± 0.0 | 1.2 ± 0.1 | 1.2 ± 0.1 | 1.2 ± 0.1 | 0.96 |
| Cholesterol, mg | 164 ± 4.0 | 165 ± 5.0 | 150 ± 5.0 | 155.9 ± 4.9 | 0.07 |  | 196 ± 4 | 200 ± 10 | 184 ± 9 | 206 ± 9 | 0.33 |
| NMES, g | 60.0 ± 1.2 | 61.7 ± 1.7 | 58.6 ± 1.7 | 59.7 ± 1.7 | 0.65 |  | 75.6 ± 1.3 | 72.8 ± 3.5 | 78.5 ± 3.1 | 69.2 ± 3.2 | 0.15 |
| Glucose, g | 14.5 ± 0.3 | 14.8 ± 0.5 | 14.6 ± 0.5 | 15.3 ± 0.5 | 0.61 |  | 18.0 ± 0.4 | 17.9 ± 1.1 | 19.2 ± 0.9 | 18.8 ± 1.9 | 0.63 |
| Sucrose, g | 44.6 ± 0.8 | 46.5 ± 1.1 | 44.2 ± 1.1 | 47.5 ± 1.1 | 0.07 |  | 50.1 ± 0.8 | 49.8 ± 2.2 | 53.8 ± 1.9 | 51.8 ± 2.0 | 0.30 |
| Maltose, g | 3.7 ± 0.1 | 3.9 ± 0.1 | 4.0 ± 0.1 | 3.8 ± 0.1 | 0.32 |  | 5.0 ± 0.1 | 5.0 ± 0.4 | 5.2 ± 0.3 | 4.5 ± 0.4 | 0.54 |
| Retinol, μg | 249 ± 15 | 267 ± 20 | 302 ± 21 | 270 ± 20 | 0.23 |  | 283 ± 15 | 255 ± 43 | 291 ± 37 | 356 ± 38 | 0.27 |
| Niacin eqv, mg | 25.9 ± 0.4 | 25.9 ± 0.5 | 27.0 ± 0.5 | 26.5 ± 0.5 | 0.30 |  | 32.6 ± 0.4 | 32.5 ± 1.1 | 32.3 ± 0.9 | 34.0 ± 1.0 | 0.55 |
| Pantothenic acid, mg | 5.1 ± 0.1 | 5.3 ± 0.2 | 5.5 ± 0.2 | 5.5 ± 0.2 | 0.10 |  | 5.6 ± 0.1 | 5.5 ± 0.3 | 5.8 ± 0.3 | 6.1 ± 0.3 | 0.34 |
| Vitamin B_6_, mg | 1.7 ± 0.0 | 1.7 ± 0.0 | 1.8 ± 0.0 | 1.7 ± 0.0 | 0.28 |  | 2.1 ± 0.0 | 2.0 ± 0.1 | 2.2 ± 0.1 | 2.2 ± 0.1 | 0.50 |
| Biotin, μg | 24.8 ± 1.2 | 26.1 ± 1.6 | 24.2 ± 1.6 | 26.0 ± 1.5 | 0.77 |  | 26.7 ± 0.8 | 26.1 ± 2.1 | 27.9 ± 1.9 | 30.1 ± 1.9 | 0.38 |
| Tryptophan/60, mg | 11.1 ± 0.2 | 11.2 ± 0.2 | 11.2 ± 0.2 | 11.7 ± 0.2 | 0.21 |  | 13.5 ± 0.1 | 14.0 ± 0.4 | 13.3 ± 0.3 | 14.4 ± 0.3 | 0.05 |
| Vitamin E, mg | 7.2 ± 0.1 | 7.1 ± 0.2 | 7.1 ± 0.2 | 6.9 ± 0.2 | 0.42 |  | 8.5 ± 0.1 | 8.4 ± 0.3 | 8.1 ± 0.2 | 8.3 ± 0.3 | 0.32 |
| Copper, mg | 0.8 ± 0.0 | 0.8 ± 0.0 | 0.8 ± 0.0 | 0.8 ± 0.0 | 0.84 |  | 0.9 ± 0.0 | 1.0 ± 0.0 | 0.9 ± 0.0 | 1.0 ± 0.0 | 0.06 |

^1^ Values shown are mean ± SEMs. Table contains nutrients that were not significantly different across NC and increasing tertiles of yogurt and fromage frais intake. NMES, non-milk extrinsic sugars; NC, non-consumer; NDNS, National Diet and Nutrition Survey; SFAs, saturated fatty acids; T, tertile.

^2^ Based on differences across non-consumers and tertiles of yogurt intake by ANCOVA controlling for age, sex and total energy intake (kJ). *Values are significantly different from non-consumers (*P*<0.05; Bonferroni post-hoc test).
