# Supplementary material for: Yogurt consumption is associated with higher nutrient intake, diet quality and favourable metabolic profile in children: a cross-sectional analysis using data from years 1–4 of the National diet and Nutrition Survey, UK
Source: Eur J Nutr. 2018 Jan 12;58(1):409–22. doi: 10.1007/s00394-017-1605-x (PMC6424923; doi:10.1007/s00394-017-1605-x)
Supplement: Supplementary file 4 — Supplementary material 4 (DOCX 104 KB) [file 394_2017_1605_MOESM4_ESM.docx]

**SUPPLEMENTAL TABLE 3** Percentage contribution to recommended nutrient intakes (RNIs) for non-yogurt consumers and across increasing tertile of yogurt and fromage frais consumption using data from years 1-4 of the NDNS for children aged 4-10 and 11-18 years old^1^

|  | **Children 4-10 y**  **(yogurt tertiles, g/d)** | | | |  | **Children 11-18 y**  **(yogurt tertiles, g/d)** | | | |
| --- | --- | --- | --- | --- | --- | --- | --- | --- | --- |
| **Component, %** | **NC**  **(0)** | **T1**  **(1-30)** | **T2**  **(31-60)** | **T3**  **(61-295)** |  | **NC**  **(0)** | **T1**  **(2-30)** | **T2**  **(31-60)** | **T3**  **(61-236)** |
| Participants, *n* | 307 | 166 | 155 | 175 |  | 610 | 97 | 89 | 88 |
| Energy | 84.0 | 87.3 | 88.9 | 91.9 |  | 77.8 | 88.8 | 80.5 | 86.4 |
| Protein | 216.3 | 224.1 | 236.0 | 248.3 |  | 139.7 | 153.6 | 144.5 | 163.4 |
| Fat, *% food energy* | 96.7 | 96.2 | 95.3 | 93.9 |  | 97.7 | 97.7 | 92.6 | 92.8 |
| Carbohydrate, *% food energy* | 103.6 | 104.1 | 104.2 | 104.3 |  | 101.0 | 102.5 | 105.3 | 103.8 |
| NMES, *% food energy* | 131.1 | 137.1 | 130.1 | 133.7 |  | 44.8 | 44.3 | 46.8 | 42.5 |
| SFA, *% food energy* | 118.2 | 120.9 | 120.6 | 121.5 |  | 114.0 | 117.4 | 110.3 | 112.9 |
| Trans fatty acids, *% food energy* | 31.2 | 31.5 | 30.3 | 31.9 |  | 31.2 | 29.8 | 30.5 | 31.1 |
| Vitamin A (retinol eqv) | 125.4 | 138.8 | 150.6 | 149.2 |  | 100.1 | 111.0 | 104.2 | 152.3 |
| Thiamin | 170.3 | 179.1 | 182.0 | 198.0 |  | 153.1 | 180.5 | 170.6 | 186.2 |
| Riboflavin | 145.5 | 159.4 | 174.8 | 192.5 |  | 114.9 | 134.5 | 129.4 | 149.9 |
| Niacin | 216.9 | 222.7 | 234.0 | 240.4 |  | 217.5 | 237.6 | 219.7 | 244.0 |
| Vitamin B_6_ | 175.8 | 180.2 | 193.9 | 190.9 |  | 166.4 | 175.5 | 178.5 | 191.5 |
| Folate | 149.8 | 153.1 | 171.0 | 167.8 |  | 101.0 | 111.5 | 110.6 | 129.4 |
| Vitamin B_12_ | 398.4 | 421.9 | 469.1 | 488.2 |  | 293.8 | 339.2 | 324.3 | 357.4 |
| Vitamin C | 270.1 | 291.1 | 274.2 | 321.2 |  | 201.0 | 249.3 | 251.4 | 272.0 |
| Potassium | 135.7 | 141.9 | 152.1 | 160.0 |  | 66.9 | 75.7 | 73.4 | 85.2 |
| Calcium | 141.1 | 154.7 | 169.4 | 188.3 |  | 82.7 | 97.7 | 91.9 | 101.2 |
| Magnesium | 116.0 | 118.7 | 128.0 | 134.8 |  | 68.4 | 78.5 | 74.3 | 87.9 |
| Iron | 110.7 | 115.1 | 117.8 | 123.9 |  | 73.5 | 80.1 | 79.6 | 90.1 |
| Copper | 114.6 | 120.1 | 123.9 | 126.7 |  | 99.8 | 116.2 | 106.9 | 116.8 |
| Zinc | 88.3 | 92.7 | 94.1 | 103.6 |  | 84.6 | 91.0 | 83.5 | 97.4 |
| Iodine | 113.2 | 125.0 | 134.5 | 155.6 |  | 86.4 | 99.6 | 101.3 | 120.8 |
| Selenium | 126.1 | 126.3 | 132.5 | 143.4 |  | 71.8 | 79.9 | 78.4 | 91.6 |

^1^Percentage difference between non-yogurt consumers and the highest tertile of yogurt consumption (T3). NME, non-milk extrinsic sugars; NC, non-consumer; NDNS, National Diet and Nutrition Survey; SFA, saturated fatty acids.
