# Supplementary material for: Yogurt consumption is associated with higher nutrient intake, diet quality and favourable metabolic profile in children: a cross-sectional analysis using data from years 1–4 of the National diet and Nutrition Survey, UK
Source: Eur J Nutr. 2018 Jan 12;58(1):409–22. doi: 10.1007/s00394-017-1605-x (PMC6424923; doi:10.1007/s00394-017-1605-x)
Supplement: Supplementary file 5 — Supplementary material 5 (DOCX 119 KB) [file 394_2017_1605_MOESM5_ESM.docx]

**SUPPLEMENTAL TABLE 4** HEI-2010 total and component scores for children aged 4-10 y and 11-18 y for non-consumers and across increasing tertile of yogurt and fromage intake using data from years 1-4 of the NDNS^1^

|  | Children 4-10 y | | | | |  | Children 11-18 y | | | | |
| --- | --- | --- | --- | --- | --- | --- | --- | --- | --- | --- | --- |
|  | Yogurt tertiles (g/d) | | | | |  | Yogurt tertiles (g/d) | | | | |
| Components | NC | T1 | T2 | T3 | *P^2^* |  | NC | T1 | T2 | T3 | *P* |
|  | (0) | (1-30) | (31-60) | (61-295) |  |  | (0) | (2-30) | (31-60) | (61-236) |  |
| Participants, n | 307 | 166 | 155 | 175 |  |  | 610 | 97 | 89 | 88 |  |
| Total HEI-2010 score (out of 100) | 57.3 ± 0.5 | 57.6 ± 0.7 | 58.9 ± 0.7 | 62.9 ± 0.6* | 0.0001 |  | 55.4 ± 0.3 | 58.6 ± 1.0* | 60.1 ± 0.8* | 66.3 ± 0.9* | 0.0001 |
| Adequacy component |  |  |  |  |  |  |  |  |  |  |  |
| Total fruit (0-5) | 3.5 ± 0.1 | 3.8 ± 0.1 | 3.6 ± 0.1 | 3.8 ± 0.1* | 0.022 |  | 2.6 ± 0.1 | 3.0 ± 0.2 | 3.2 ± 0.2* | 3.4 ± 0.2* | 0.0001 |
| Whole fruit (0-5) | 3.1 ± 0.1 | 3.4 ± 0.1 | 3.3 ± 0.1 | 3.6 ± 0.1* | 0.004 |  | 1.7 ± 0.1 | 2.3 ± 0.2* | 2.3 ± 0.2* | 3.1 ± 0.2* | 0.0001 |
| Total vegetables (0-5) | 1.0 ± 0.0 | 1.0 ± 0.0 | 1.0 ± 0.0 | 1.0 ± 0.0 | 0.57 |  | 1.0 ± 0.0 | 1.0 ± 0.0 | 1.0 ± 0.0 | 1.0 ± 0.0 | 0.48 |
| Greens beans (0-5) | 2.2 ± 0.1 | 2.3 ± 0.1 | 2.1 ± 0.1 | 2.2 ± 0.1 | 0.59 |  | 1.8 ± 0.1 | 1.9 ± 0.1 | 2.0 ± 0.1 | 2.4 ± 0.1* | 0.0001 |
| Wholegrain (0-10) | 5.2 ± 0.2 | 4.9 ± 0.3 | 5.2 ± 0.3 | 5.0 ± 0.3 | 0.82 |  | 4.1 ± 0.2 | 4.4 ± 0.4 | 4.9 ± 0.4 | 5.9 ± 0.4* | 0.0001 |
| Dairy portions (0-10) | 3.2 ± 0.1 | 4.5 ± 0.1* | 6.1 ± 0.1* | 8.4 ± 0.1* | 0.0001 |  | 2.5 ± 0.1 | 3.7 ± 0.2* | 4.7 ± 0.2* | 7.3 ± 0.2* | 0.0001 |
| Sodium (0-10) | 5.0 ± 0.2 | 4.9 ± 0.2 | 4.5 ± 0.2 | 5.1 ± 0.2 | 0.30 |  | 8.4 ± 0.1 | 8.4 ± 0.2 | 8.7 ± 0.2 | 8.8 ± 0.2 | 0.12 |
| Seafood & plant proteins (0-5) | 2.3 ± 0.1 | 2.2 ± 0.2 | 2.0 ± 0.2 | 2.5 ± 0.2 | 0.12 |  | 1.6 ± 0.1 | 2.0 ± 0.2 | 2.0 ± 0.2 | 2.6 ± 0.2* | 0.0001 |
| Total protein foods (0-5) | 1.5 ± 0.1 | 1.6 ± 0.1 | 1.7 ± 0.1 | 1.7 ± 0.1 | 0.58 |  | 1.9 ± 0.1 | 1.9 ± 0.2 | 1.9 ± 0.2 | 1.8 ± 0.2 | 0.99 |
| Moderation component |  |  |  |  |  |  |  |  |  |  |  |
| Refined grains (0-5) | 4.6 ± 0.2 | 3.5 ± 0.3* | 3.7 ± 0.3 | 4.4 ± 0.3 | 0.001 |  | 4.2 ± 0.2 | 4.3 ± 0.4 | 4.4 ± 0.4 | 4.4 ± 0.4 | 0.94 |
| Empty calories (0-20) | 19.4 ± 0.1 | 19.4 ± 0.1 | 19.6 ± 0.1 | 19.5 ± 0.1 | 0.69 |  | 18.7 ± 0.1 | 18.9 ± 0.3 | 18.3 ± 0.3 | 19.1 ± 0.3 | 0.22 |
| Fatty acid ratio (0-10) | 6.4 ± 0.1 | 6.1 ± 0.1 | 6.1 ± 0.1 | 5.8 ± 0.1* | 0.0001 |  | 7.0 ± 0.1 | 6.8 ± 0.2 | 6.7 ± 0.2 | 6.5 ± 0.2 | 0.024 |

^1^ Values shown are mean ± SEMs after adjustment. Higher HEI-2010 score is associated with better diet quality. HEI-2010, Healthy Eating Index-2010; NC, non-consumer; National Diet and Nutrition Survey, NDNS; T, tertile.

^2^ Based on differences across non-consumers and tertiles of yogurt intake by ANCOVA controlling for age, sex and total energy intake (kJ).* Values are significantly different from non-consumers (*P*<0.05; Bonferroni post-hoc test).
