# Supplementary material for: Yogurt consumption is associated with higher nutrient intake, diet quality and favourable metabolic profile in children: a cross-sectional analysis using data from years 1–4 of the National diet and Nutrition Survey, UK
Source: Eur J Nutr. 2018 Jan 12;58(1):409–22. doi: 10.1007/s00394-017-1605-x (PMC6424923; doi:10.1007/s00394-017-1605-x)
Supplement: Supplementary file 6 — Supplementary material 6 (DOCX 26 KB) [file 394_2017_1605_MOESM6_ESM.docx]

**Supplemental Table 5** Metabolic profiles of children aged 4-10 y and 11-18 y for non-consumers and increasing tertile of yogurt and fromage frais consumption using data from years 1-4 of the NDNS^1^

|  | Children 4-10 y  Yogurt tertiles (g/d) | | | |  |  | Children 11-18 y  Yogurt tertiles (g/d) | | | |  |
| --- | --- | --- | --- | --- | --- | --- | --- | --- | --- | --- | --- |
| Biomarkers | NC  (0) | T1  (1-30) | T2  (31-60) | T3  (61-295) | *P^2^* |  | NC  (0) | T1  (2-30) | T2  (31-60) | T3  (61-236) | *P* |
| Participants, n | 307 | 166 | 155 | 175 |  |  | 610 | 97 | 89 | 88 |  |
| Height, m |  |  |  |  |  |  |  |  |  |  |  |
| Model 2^3^ | 124.9 ± 0.4 | 124.6 ± 0.5 | 125.0 ± 0.5 | 125.4 ±0.5 | 0.71 |  | 163.8 ± 0.1 | 163.7 ± 0.9 | 165.9 ± 0.8 | 162.7 ± 0.8 | 0.033 |
| Model 3^4^ | 124.9 ± 0.4 | 124.7 ± 0.5 | 125.0 ± 0.5 | 125.3 ± 0.5 | 0.83 |  | 163.7 ± 0.3 | 163.7 ± 1.0 | 166.0 ± 0.8 | 162.9 ± 0.9 | 0.035 |
| Weight, kg |  |  |  |  |  |  |  |  |  |  |  |
| Model 2 | 27.4 ± 0.2 | 27.2 ± 0.2 | 27.4 ± 0.3 | 27.5 ± 0.2 | 0.88 |  | 59.3 ± 0.2 | 59.4 ± 0.7 | 60.7 ± 0.6 | 58.8 ± 0.6 | 0.09 |
| Model 3 | 27.4 ± 0.2 | 27.2 ± 0.3 | 27.5 ± 0.3 | 27.5 ± 0.3 | 0.90 |  | 59.2 ± 0.2 | 59.4 ± 0.7 | 60.8 ± 0.6 | 59.0 ± 0.6 | 0.09 |
| BMI, kg/m^2^ |  |  |  |  |  |  |  |  |  |  |  |
| Model 2 | 17.2 ±0.2 | 17.1 ± 0.2 | 16.9 ± 0.2 | 17.3 ± 0.2 | 0.59 |  | 21.9 ± 0.2 | 22.2 ± 0.5 | 21.9 ± 0.4 | 22.0 ± 0.5 | 0.95 |
| Model 3 | 17.2 ± 0.2 | 17.1 ± 0.2 | 16.9 ± 0.2 | 17.3 ± 0.2 | 0.62 |  | 21.8 ± 0.2 | 22.2 ± 0.5 | 21.9 ± 0.4 | 22.2 ± 0.5 | 0.84 |
| Waist, cm |  |  |  |  |  |  |  |  |  |  |  |
| Model 2 | N/A | N/A | N/A | N/A | N/A |  | 76.3 ± 0.3 | 75.9 ± 0.8 | 76.8 ± 0.6 | 74.9 ± 0.7 | 0.18 |
| Model 3 | N/A | N/A | N/A | N/A | N/A |  | 76.2 ± 0.3 | 75.9 ± 0.8 | 76.9 ± 0.6 | 75.1 ± 0.7 | 0.28 |
| Hip, cm |  |  |  |  |  |  |  |  |  |  |  |
| Model 2 | N/A | N/A | N/A | N/A | N/A |  | 93.9 ± 0.2 | 93.9 ± 0.6 | 95.7 ± 0.5 | 93.5 ± 0.5 | 0.009 |
| Model 3 | N/A | N/A | N/A | N/A | N/A |  | 94.0 ± 0.2 | 93.9 ± 0.6 | 95.7 ± 0.5 | 93.3 ± 0.6 | 0.008 |
| Waist:Hip ratio |  |  |  |  |  |  |  |  |  |  |  |
| Model 2 | N/A | N/A | N/A | N/A | N/A |  | 0.8 ± 0.0 | 0.8 ± 0.0 | 0.8 ± 0.0 | 0.8 ± 0.0 | 0.37 |
| Model 3 | N/A | N/A | N/A | N/A | N/A |  | 0.8 ± 0.0 | 0.8 ± 0.0 | 0.8 ± 0.0 | 0.8 ± 0.0 | 0.67 |
| SBP, mm Hg |  |  |  |  |  |  |  |  |  |  |  |
| Model 2 | 103.9 ± 0.6 | 103.8 ± 0.9 | 103.5 ± 0.9 | 104.7 v 0.8 | 0.80 |  | 112.8 ± 0.5 | 111.3 ± 1.4 | 113.5 ± 1.2 | 115.0 ± 1.2 | 0.20 |
| Model 3 | 103.8 ± 0.7 | 103.7 ± 0.9 | 103.5 ± 0.9 | 105.1 ± 0.8 | 0.52 |  | 112.7 ± 0.5 | 111.4 ± 1.4 | 113.6 ± 1.0 | 115.4 ± 1.3 | 0.13 |
| DBP, mm Hg |  |  |  |  |  |  |  |  |  |  |  |
| Model 2 | 63.0 ± 0.6 | 63.9 ± 0.8 | 61.9 ± 0.9 | 62.7 ± 0.8 | 0.46 |  | 63.4 ± 0.4 | 63.6 ± 1.2 | 63.5 ± 0.9 | 63.6 ± 0.9 | 0.99 |
| Model 3 | 62.8 ± 0.6 | 63.7 ± 0.8 | 62.0 ± 0.9 | 63.2 ± 0.8 | 0.53 |  | 63.4 ± 0.4 | 63.6 ± 1.2 | 63.5 ± 1.0 | 63.6 ± 1.0 | 0.99 |
| PP, mm Hg |  |  |  |  |  |  |  |  |  |  |  |
| Model 2 | 87.6 ± 0.7 | 85.8 ± 0.9 | 84.6 ± 0.9 | 84.4 ± 0.9 | 0.024 |  | 74.6 ± 0.5 | 74.9 ± 1.4 | 74.4 ± 1.2 | 72.5 ± 1.2 | 0.39 |
| Model 3 | 87.5 ± 0.7 | 85.7 ± 0.9 | 84.6 ± 0.9 | 84.5 ±0.9 | 0.038 |  | 74.5 ± 0.5 | 75.0 ± 1.4 | 74.6 ± 1.2 | 73.0 ± 1.3 | 0.70 |
| Cholesterol, mmol/L |  |  |  |  |  |  |  |  |  |  |  |
| Model 2 | 4.5 ± 0.1 | 4.3 ± 0.1 | 4.2 ± 0.1 | 4.5 ± 0.1 | 0.16 |  | 4.1 ± 0.1 | 4.1 ± 0.1 | 3.9 ± 0.1 | 3.9 ± 0.1 | 0.19 |
| Model 3 | 4.5 ± 0.1 | 4.3 ± 0.1 | 4.2 ± 0.1 | 4.5 ± 0.1 | 0.16 |  | 4.1 ± 0.1 | 4.1 ± 0.1 | 3.9 ± 0.1 | 3.8 ± 0.1 | 0.15 |
| HDL-C, mmol/L |  |  |  |  |  |  |  |  |  |  |  |
| Model 2 | 1.6 ± 0.0 | 1.5 ± 0.1 | 1.6 ± 0.1 | 1.6 ± 0.1 | 0.60 |  | 1.4 ± 0.0 | 1.4 ± 0.1 | 1.4 ± 0.0 | 1.4 ± 0.1 | 0.99 |
| Model 3 | 1.6 ± 0.0 | 1.5 ± 0.1 | 1.6 ± 0.1 | 1.6 ± 0.1 | 0.61 |  | 1.4 ± 0.0 | 1.4 ± 0.1 | 1.4 ± 0.1 | 1.4 ± 0.1 | 0.99 |
| LDL-C, mmol/L |  |  |  |  |  |  |  |  |  |  |  |
| Model 2 | 2.6 ± 0.1 | 2.5 ± 0.1 | 2.3 ± 0.1 | 2.6 ± 0.1 | 0.21 |  | 2.3 ± 0.0 | 2.4 ± 0.1 | 2.1 ± 0.1 | 2.2 ± 0.1 | 0.21 |
| Model 3 | 2.6 ± 0.1 | 2.5 ± 0.1 | 2.3 ± 0.1 | 2.6 ± 0.1 | 0.21 |  | 2.3 ± 0.0 | 2.4 ± 0.1 | 2.1 ± 0.1 | 2.1 ±0.1 | 0.18 |
| HDL ratio |  |  |  |  |  |  |  |  |  |  |  |
| Model 2 | 2.8 ± 0.1 | 2.9 ± 0.1 | 2.6 ± 0.1 | 2.8 ± 0.1 | 0.23 |  | 3.0 ± 0.1 | 3.0 ± 0.1 | 2.8 ± 0.1 | 2.8 ± 0.1 | 0.30 |
| Model 3 | 2.9 ± 0.1 | 2.9 ± 0.1 | 2.6 ± 0.1 | 2.8 ± 0.1 | 0.23 |  | 3.0 ± 0.1 | 3.0 ± 0.1 | 2.8 ± 0.1 | 2.8 ± 0.1 | 0.41 |
| TAG, mmol/L |  |  |  |  |  |  |  |  |  |  |  |
| Model 2 | 0.8 ± 0.1 | 0.7 ± 0.1 | 0.6 ± 0.1 | 0.7 ± 0.1 | 0.37 |  | 0.9 ± 0.0 | 0.8 ± 0.1 | 0.8 ± 0.1 | 0.8 ± 0.1 | 0.82 |
| Model 3 | 0.8 ± 0.1 | 0.7 ± 0.1 | 0.6 ± 0.1 | 0.7 ± 0.1 | 0.37 |  | 0.8 ± 0.0 | 0.9 ± 0.1 | 0.8 ± 0.1 | 0.8 ± 0.1 | 0.98 |
| CRP, mg/L |  |  |  |  |  |  |  |  |  |  |  |
| Model 2 | 1.7 ± 0.3 | 1.4 ± 0.4 | 2.5 ± 0.4 | 1.5 ± 0.4 | 0.15 |  | 1.7 ± 0.1 | 1.8 ± 0.2 | 1.5 ± 0.2 | 1.7 ± 0.2 | 0.68 |
| Model 3 | 1.7 ± 0.3 | 1.3 ± 0.4 | 2.5 ± 0.4 | 1.7 ± 0.4 | 0.15 |  | 1.7 ± 0.1 | 1.8 ± 0.2 | 1.5 ± 0.2 | 1.6 ± 0.2 | 0.60 |
| HbA1c, mmol/L |  |  |  |  |  |  |  |  |  |  |  |
| Model 2 | 5.3 ± 0.0 | 5.3 ± 0.0 | 5.2 ± 0.0 | 5.3 ± 0.0 | 0.85 |  | 5.3 ± 0.0 | 5.3 ± 0.1 | 5.3 ± 0.1 | 5.1 ± 0.1 | 0.005 |
| Model 3 | 5.3 ± 0.0 | 5.3 ± 0.0 | 5.3 ± 0.1 | 5.3 ± 0.0 | 0.84 |  | 5.3 ± 0.0 | 5.3 ± 0.1 | 5.3 ± 0.1 | 5.1 ± 0.1 | 0.010 |
| Glucose, mmol/L |  |  |  |  |  |  |  |  |  |  |  |
| Model 2 | 4.8 ± 0.1 | 4.7 ± 0.1 | 4.7 ± 0.1 | 4.7 ± 0.1 | 0.63 |  | 4.8 ± 0.0 | 4.8 ± 0.1 | 4.8 ± 0.1 | 4.6 ± 0.1 | 0.26 |
| Model 3 | 4.8 ± 0.1 | 4.8 ± 0.1 | 4.7 ± 0.1 | 4.7 ± 0.1 | 0.48 |  | 4.8 ± 0.0 | 4.8 ± 0.1 | 4.8 ± 0.1 | 4.7 ± 0.1 | 0.45 |

^1^ Values are mean ± SEMs of adjusted values. CRP, C-reactive protein; DBP, diastolic blood pressure; HbA1c, glycated haemoglobin; NDNS, national diet and nutrition survey; NC, non-consumer; N/A, not available; PP, pulse pressure; SBP, systolic blood pressure; TAGs, Triacylglycerols; T, tertile.

^2^ Based on differences across non-consumers and tertiles of yogurt intake by ANCOVA

^3^ Model 2 adjusted for age, sex, BMI and total energy intake (kJ/d).

^4^ Model 3 additional adjustment for HEI-2010 score.
